# Multiple Layers of Stress-Induced Regulation in tRNA Biology

**DOI:** 10.3390/life6020016

**Published:** 2016-03-23

**Authors:** Hsiao-Yun Huang, Anita K. Hopper

**Affiliations:** 1 Department of Biology, Indiana University, 915 E third St., Myers 300, Bloomington, IN 47405, USA; 2 Department of Molecular Genetics and Center for RNA Biology, The Ohio State University, Columbus, OH 43210, USA; hopper.64@osu.edu

**Keywords:** tRNA processing, tRNA subcellular dynamics, tRNA modification, tRNA fragments

## Abstract

tRNAs are the fundamental components of the translation machinery as they deliver amino acids to the ribosomes during protein synthesis. Beyond their essential function in translation, tRNAs also function in regulating gene expression, modulating apoptosis and several other biological processes. There are multiple layers of regulatory mechanisms in each step of tRNA biogenesis. For example, tRNA 3′ trailer processing is altered upon nutrient stress; tRNA modification is reprogrammed under various stresses; nuclear accumulation of tRNAs occurs upon nutrient deprivation; tRNA halves accumulate upon oxidative stress. Here we address how environmental stresses can affect nearly every step of tRNA biology and we describe the possible regulatory mechanisms that influence the function or expression of tRNAs under stress conditions.

## 1. Introduction

Transfer RNAs (tRNAs) serve their essential roles of delivering amino acids to the cytoplasmic protein synthesis machinery. To function as an adaptor during protein synthesis, the tRNA isoacceptor is charged with a cognate amino acid to form aminoacyl-tRNA (aa-tRNA). Beyond the essential role of translating the genetic code, tRNAs also function in other biological processes, such as signaling in the general amino acid control (GAAC) pathway, sensing the nutritional stress, serving as primers for retroviral replication, and regulation of apoptosis by directly binding to cytochrome C [1,2,3,4,5]. Aminoacylated tRNAs have also been implicated as substrates for non-ribosomal peptide bond formation, modification of phospholipids in the cell membrane, protein labeling for degradation, and antibiotic biosynthesis [5]. Therefore, tRNAs have emerging roles in adaptive translation, signaling dynamics, and regulating biological processes [6,7,8].

tRNA biogenesis involves multiple steps, many of which are conserved throughout eukaryotes. Eukaryotic tRNAs are transcribed by RNA polymerase III as precursor molecules (pre-tRNAs). The pre-tRNAs undergo an elaborate set of post-transcriptional steps to generate mature tRNAs. These steps include: removal of both the 5′ leader and 3′ trailer sequences, nucleotide addition to all 3′ ends and to a 5′ end of one tRNA, tRNA^His^, removal of introns from transcripts transcribed by intron-containing genes, and addition of nucleotide modifications. In addition to the regulation of each step of tRNA biogenesis, cells also employ multiple tRNA quality control mechanisms to prevent inappropriate substrates being used in the protein synthesis [9]. Due to the importance of functional tRNA, alterations in the levels of tRNA transcripts and defects in tRNA processing and modifications result in several human diseases, including neuronal disorders [10], pontocerebellar hypoplasia [11], nonsyndromic X-linked intellectual disability (NSXLID) [12] and cancer [6,7,13,14,15,16].

**Figure 1:**
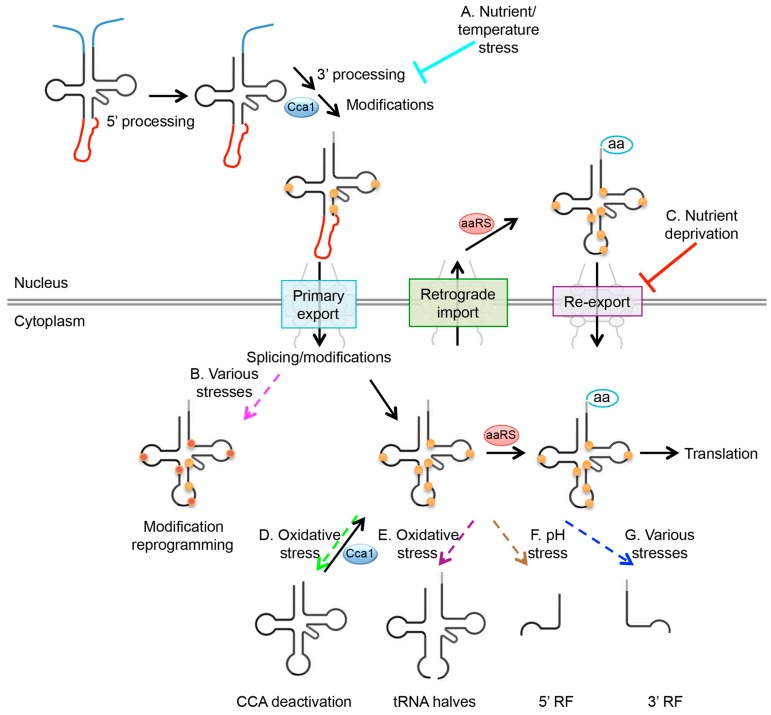
Stress-induced regulation in tRNA biology. The canonical tRNA biogenesis pathway and subcellular traffic are indicated with solid black arrows. (**A**) Upon nutrient and/or temperature stress, 3′ trailer sequence processing is likely inhibited and thus aberrant pre-tRNAs accumulate (Cyan blunt-ending line); (**B**) tRNA modification is subject to change under certain cellular conditions (Magenta dotted arrow); (**C**) Upon nutrient deprivation, tRNA re-export step is likely inhibited by multiple mechanisms and thus tRNAs accumulate in the nucleus (Red blunt-ending line); (**D**) Upon oxidative stress, tRNAs are endonucleolytically cleaved within their 3′ CCA termini (Green dotted arrow). Oxidative stress-induced deactivation of the 3′ CCA termini is a dynamically reversible process; (**E**) When tRNAs are exposed to oxidative stress, heat shock, and UV irradiation, mature tRNAs are endonucleolytically cleaved in the anticodon loops, generating 5′ and 3′ tRNA halves (Purple dotted arrow); (**F**) The 5′ tRFs are derived from the 5′ parts of mature tRNAs and are formed by a cleavage in the D loop. 5′ tRF can be formed when cells are grown at high pH environment (Brown dotted arrow); (**G**) 3′ CCA tRFs correspond to 3′ parts of mature tRNAs containing processed 3′ CCA termini and are formed by cleavage at the T loop (Blue dotted arrow). Dicer, angiogenin, and other RNase A family members have been implicated in the generation of the 3′ tRF.

tRNAs are stable with half-lives estimated from ~9 h to up to days [16,17,18]. As tRNA biogenesis involves multiple steps, the expression and/or function of tRNA is likely to be tightly regulated by distinct pathways. Several reviews have discussed the modulation of tRNA levels and modifications in controlling translation [6,8,19]. Here we address the data supporting the notion that environmental stresses can influence nearly every step of tRNA biology. We focus on the regulation of tRNA processing, modification, subcellular dynamics, and fragmentation that are altered in response to stress conditions (Figure 1). This review focuses on the recent discoveries employing the yeast, *Saccharomyces cerevisiae*. In addition, similarities and differences of the regulatory mechanisms in budding yeast to those in other organisms are described if the information is available.

## 2. Pre-tRNA Processing

The majority of yeast pre-tRNA transcripts contain 5′ leader and 3′ trailer sequences [20,21]. Maturation of tRNAs in nearly all organisms begins with removal of the 5′ extension by the endonuclease RNase P which is located in the nucleolus in yeast [22]. In bacteria, Archaea, fungi and vertebrates, RNase P, is a ribonucleoprotein complex comprised of a single RNA and a variable number of protein subunits [23], but in plants mitochondrial and nuclear forms of RNase P are protein-only enzymes [24,25]. The RNA subunit is the catalytic component in bacteria which have one protein subunit [26,27] and in Archaea which have five protein components [28,29]. As the RNA component of human RNase P is catalytic but with very poor kinetics, the eukaryotic protein subunits have been proposed to play a supportive role in catalyzing the removal of the 5′ end of tRNA [30,31,32]. Yeast nucleolar RNase P consists of nine proteins (Pop1, Pop3-8, Rpp1, and Rpr2) and a single essential RNA (RPR1) [22]. The source of eukaryotic RNase P catalysis remains unclear since RNA components have substantial differences in regions important for stability and catalysis [30,31]. In addition to the canonical role in pre-tRNA processing, most of the proteins subunits of the yeast and human RNase P are shared with RNase MRP, involved in rRNA maturation [22,23,25].

3′ end maturation is far more complex than 5′ end processing of tRNA. Maturation of 3′ extensions from pre-tRNA requires both exo- and endo-nucleases [16,33,34]. Yeast Rex1 is a 3′ to 5′ exonuclease that participates in the processing of pre-tRNA trailers as well as in the processing of other RNAs such as 5S rRNA, 5.8S rRNA, and snRNAs [35,36,37]. RNase Z, yeast Trz1, is the endonuclease that participates in 3′ end processing for both mitochondrial and nuclear encoded tRNAs [4,34,38,39,40]. Recent studies show that the majority of tRNAs utilize both endonucleolytic cleavage and exonucleolytic trimming pathways, with a preference for the endonucleolytic cleavage [34]. Exonucleolytic digestion occurs to some degree in wild-type cells and not only when endonucleolytic cleavage is inhibited. However, 3′ end processing of pre-tRNAs with longer 3′ trailers depends to a greater extent on endonuclease, probably due to an inefficient exonucleolytic pathway, inhibited by secondary structures comprised within their longer 3′ extension [34]. The tRNA binding La protein (yeast Lhp1) is involved in pre-tRNA processing. Lhp1 that possesses chaperon activity stabilizes pre-tRNAs by directly binding to the oligo U within 3′ termini, which are generated upon Pol III termination [41,42]. Precursors with different 3′ ends display diverse affinities for La binding, which favors Trz1-mediated endonucleolytic cleavage and protects against exonucleolytic trimming [43]. It is proposed that 3′ oligo U length is a primary determinant of La binding with subsequent steps distinguished by 3′ endo- *vs.* exo-nuclease, chaperon activities and nuclear surveillance [35,40,44,45].

## 3. tRNA 3′ Processing Can Be Regulated by Stress

Recent studies suggest that in budding yeast 3′ processing of pre-tRNAs is specifically regulated by growth conditions [46]. Analyses of Northern blot and RNA sequencing show that upon shift to elevated temperatures and/or to glycerol-containing medium, aberrant pre-tRNAs accumulate in a tRNA-specific manner in yeast. Under heat stress elevated levels of aberrant tRNA^Tyr^ with extended 3′ termini and processed 5′ ends are detected, suggesting that 3′ trailer removal of tRNA^Tyr^ is inhibited (Figure 1A). Interestingly, stress-induced inhibition of pre-tRNA^Ile^_UAU_ processing results in accumulation of unprocessed pre-tRNA^Ile^_UAU_ at both 3′ and 5′ termini and part of these pre-tRNAs with longer 3′ trailers. As cells with deletion of *REX1* accumulated 3′ extended pre-tRNAs even under normal conditions, yeast Rex1 nuclease appears to be limiting for 3′ end processing [46]. It is possible that Rex1 and other 3′ processing enzymes are regulated by cell growth conditions to influence 3′ processing of pre-tRNAs or the levels and stability of pre-tRNAs are altered upon stress. Although tRNA 3′ processing pathway is conserved, whether stress-induced inhibition of 3′ processing occurs in vertebrates requires further investigations.

Human La protein is regulated by growth conditions [47]. Under nutrient-rich conditions, La is phosphorylated and localized in the nucleoplasm where it can associate with pre-tRNAs [48,49]. In contrast, non-phosphorylated La distributes throughout the cells and it preferentially associated with 5′-terminal oligopyrimidine-containing mRNAs that control protein synthesis in the cytoplasm [49]. After induction of apoptosis by exposing cells to various types of DNA damage reagents, La is rapidly dephosphorylated by a protein phosphatase 2A-like activity and a subset of La is proteolytically cleaved *in vivo* [50]. Therefore, post-translational modification of La provides a mechanism to regulate tRNA processing.

## 4. tRNA Nucleotidyl Transferase in Stress Response

All tRNAs contain a 3′ terminal CCA sequence that is necessary for tRNA aminoacylation. In yeast and vertebrates, addition of 3′ CCA sequence is catalyzed by nucleotidyl transferase, while *E. coli* tRNAs are encoded with a CCA sequence. But *E. coli* also possesses the gene for the CCA adding enzyme, which functions in tRNA 3′ end repair [51,52]. Yeast tRNA nucleotidyl transferase is encoded by *CCA1* [53]. *CCA1* encodes multiple isoforms, Cca1-I, Cca1-II, and Cca1-III, which are generated by alternative transcriptional and distinct translational start sites. These isoforms are differently distributed among mitochondria, the cytoplasm, and the nucleoplasm [54]. Addition of 3′ CCA sequence is normally catalyzed by the nuclear pool of CCA adding enzyme, while the cytoplasmic pool functions in tRNA 3′ repair. It is thought that the mitochondrial form functions in both biogenesis and 3′ end repair of mitochondria encoded tRNAs.

Recent data for vertebrate cells have shown that upon oxidative stress the 3′ CCA sequence is removed by endonuclease angiogenin, thereby rapidly repressing translation. Upon removal of stress addition of 3′ CCA ends is restored by nucleotidyl transferase, allowing aminoacylation and translation to ensue [55]. Therefore, deactivation of CCA sequence of tRNA leads to a rapid and reversible regulation of translation repression upon stress. tRNA nucleotidyl transferase can generate 3′ CCACCA termini via extended polymerization on particular tRNAs that are hypomodified and/or possess aberrant tertiary conformations, and these CCACCA-containing tRNAs are subsequently targeted for degradation [56,57]. Therefore, tRNA nucleotidyl transferase is responsive to stress to regulate translation and control tRNA quality (Figure 1D).

## 5. tRNA Modifications

One of the remarkable features of tRNA from all organisms is that they are highly modified by numerous post-transcriptional steps. More than 100 different modifications are known in nature, ranging from single methylation to multiple step reactions [58]. Some modifications are restricted to Archaea, bacteria, or eukaryotes, but many are shared. Some modified bases are present in almost all tRNAs, such as dihydrouridine (D) and pseudouridine (Ψ), whereas others are present only on a single tRNA [59,60].

To generate complexity of highly modified tRNA isoacceptors, genomes encode a large number of enzymes responsible for catalyzing the correct modifications at the proper site of particular tRNAs. The majority of the genes that are responsible for modifications have been identified in *S. cerevisiae*, and a large number of genes for tRNA modification have been identified in other organisms and compiled at Modomics (http://modomics.genesilico.pl) [16].

It is now know that tRNA modifications serve numerous purposes, including translation fidelity, maintenance of proteome integrity, tRNA folding or stability, and tRNA discrimination. In general, many modifications in or around the anticodon loop affect translation or growth by regulating codon-anticodon interactions and reading frame maintenance. The deamination of adenosine (A) to inosine (I) at wobble position 34 of tRNA provides an example of tRNA modification affecting decoding. As A only base pairs with U, but I base pairs with U, C, and A, tRNAs with I at the wobble position have a broader codon-anticodon interaction capacity [61]. Modifications in the anticodon loop can also maintain the reading frames during translation. For example, mutation of the genes responsible for (yW) modification of tRNA^Phe^ at position 37 results in increases in -1 frameshifting during translation [62].

Interestingly, modifications at wobble base can maintain proteome integrity. In eukaryotes, the wobble uridine (U_34_) base of tRNAs carries a 5-methoxycarbonylmethyl (mcm^5^) or 5-carbamoylmethyl (ncm^5^). Following mcm^5^U_34_ addition, base of tRNA^Glu^_UUC_, tRNA^Lys^_UUU_, and tRNA^Gln^_UUG_ is further decorated with a 2-thio group (s^2^) [63,64,65]. As loss of U_34_ modifications leads to ribosome pausing at their cognate codons in yeast and *Caenorhabditis elegans*, U_34_ modifications affect the translation rates at a subset of cognate codons. Cells lacking U_34_ modifications trigger proteotoxic stress and accumulate aggregates of endogenous proteins and therefore result in an imbalance in protein homeostasis [66]. In addition, an integrated analysis of proteome, transcriptome and gene-specific ribosome footprinting indicate that Trm9-catalyzed tRNA modifications at the wobble position, mcm^5^U and mcm^5^s^2^U, regulate global protein expression via codon-biased translation [67].

Modifications in the main body of the tRNA can affect tRNA folding or stability. For example, reduced levels of tRNA^Ser^_CGA_ are observed in strains with a tRNA^Ser^ mutation that also lack m^5^U_54_ or Ψ_55_ due to deletion of *TRM2* or *PUS4* [68] and reduced levels of tRNA^Ser^_CGA_ and tRNA^Ser^_UGA_ are detected at high temperature in strains lacking Um_44_ and ac^4^C_12_ due to deletion of *TRM44* and *TAN1* [69,70]. Although tRNAs are among the most stable RNA species *in vivo*, they can be degraded when the sequence or modifications of the tRNAs are altered. In addition, when cells possess mutations of multiple modifications genes, such as *pus1*Δ *pus4*Δ, *trm4*Δ *trm8*Δ, or *trm44*Δ *tan1*Δ synthetic lethality or temperature sensitive growth phenotype occurs [71,72]. The temperature sensitive growth is likely caused by tRNA instability [70,73], and turnover of unstable tRNA is mediated by the 5′ to 3′ rapid tRNA decay pathway (RTD) [72]. Thus, tRNA modifications are key determinants of tRNA stability.

Modifications at various positions specifically provide tRNA discrimination. For instance, tRNA_i_^Met^ is modified at adenosine 64 (Ar(p)64) by Rit1 which only interacts with unique T stem-loop of tRNA_i_^Met^. Modified tRNA_i_^Met^ does not interact with eEF1A so that it functions only at an initiating AUG codon [74]. However, the exact functions of many of other modifications are not yet known.

## 6. Reprogramming of tRNA Modification upon Stress

tRNA modification enzymes are misregulated in various human diseases, including cancer and neurological disorders, suggesting tRNA modification is subject to change under certain cellular conditions [7,47]. Several studies provide evidence that tRNA modifications are altered under elevated temperature and growth arrest conditions [75,76,77,78]. Recent high-throughput modification analyses by using a chromatography-coupled mass spectrometry platform have revealed that the levels of assessed tRNA modification are uniquely changed in response to H_2_O_2_, methyl methanesulfonate (MMS), arsenite, and hypochlorite [79] (Figure 1B). Chan *et al.* reported that the levels of 23 of the known 25 ribonucleoside modifications individually altered in response to each toxicant with signature changes in the hierarchical clustering analyses [47,79]. Pseudouridine, the most abundant RNA modification, is regulated by cell growth state and temperature as Ψ-seq profiles show that the levels of pseudouridine modification on mRNA and ncRNA vary upon environmental change [80,81]. Altered tRNA modifications may affect the stability of specific tRNAs, causing these tRNAs to be degraded or stabilized under certain cellular conditions. In addition, these stress-specific patterns of tRNA modification changes are linked to selective translation of codon-biased mRNAs for stress response proteins [19,47]. For example, *in S. cerevisiae*, the levels of 5-methylcytosine (m^5^C) modification by tRNA methyltransferase, Trm4, at the wobble C_34_ base in tRNA^Leu^_CAA_ increase upon exposure to oxidative stress. This altered modification allows selective translation of stress-related genes with over-represented Leu-UUG codons, including the ribosomal protein Rpl22a [82]. In contrast, the levels of m^5^C are largely unaffected upon exposure to other stresses, such as MMS, arsenite, and hypochlorite. 

Changes in wobble base tRNA modification levels may influence codon usage patterns in specific transcripts, designated as modification tunable transcripts (MoTTs), which is codon specific regulation of translation [83]. Chan *et al.* demonstrated a coordinated translational stress response system involving stress-specific reprogramming of tRNA wobble modifications that leads to selective translation of codon-biased mRNAs representing different classes of critical response proteins [84]. Therefore, distinct stresses uniquely reprogram tRNA modifications to modulate certain transcripts and control translation, suggesting that alteration of tRNA modifications serves as a novel regulatory mechanism for translational control in stress response.

tRNA m^5^C methylatransferase Dnmt2 also has a prominent role in the stress response. *Drosophila Dnmt2* mutants are significantly less viable under oxidative or heat stress conditions, and Dnmt2 relocalized to stress granules following heat shock. Substrate tRNAs in *Dnmt2* mutants are more sensitive to stress-induced angiogenin-mediated cleavage, suggesting that Dnmt2-mediated tRNA methylation may play a role in protecting tRNAs from stress-induced cleavage [85]. In contrast, the *Kluyveromyces lactis* γ-toxin is a secreted endonuclease that inhibits growth of sensitive microbes, such as *S. cerevisiae*. γ-toxin cleaves tRNAs that possess mcm^5^s^2^U modification at the wobble position [86]. Therefore, tRNA anticodon loop modifications can influence tRNA endonucleolytic cleavage.

## 7. tRNA Subcellular Dynamics

tRNA subcellular trafficking was considered as a one-way movement, from the nucleus, the site of tRNA biogenesis, to the cytoplasm, the site of protein synthesis. However, it is now known that tRNA subcellular movement is bidirectional between the nucleus and the cytoplasm. Newly transcribed end-processed tRNAs move from the nucleus to the cytoplasm via primary nuclear export step. The cytoplasmic tRNAs travel back to the nucleus via retrograde nuclear import and the imported tRNAs then once again access the cytoplasm via the re-export step (Reviews: [4,9,16,87]). The tRNA retrograde pathway is conserved from yeast to vertebrates [88]. This retrograde pathway serves for at least four roles in the distinct cellular processes, including yW_37_ modification of yeast tRNA^Trp^ [89], delivery of retrotranscirbed HIV genomes to the nucleus [90], tRNA quality control [9,91], and efficient translation of particular mRNAs involved in methionine and arginine biosynthesis [92].

Movement of tRNAs between the nucleus and the cytoplasm proceeds via association of importin-β family members. In vertebrates, importin-β family member Exportin-t functions in tRNA nuclear export [93,94]. Homologs of exportin-t have been studied in budding yeast (Los1), fission yeast (Xpo-t), and plants (PAUSED) [95,96,97,98,99]. *In vitro* biochemical studies of the vertebrate exportin-t and crystallography structural studies of the fission yeast Xpo-t show that exportin-t preferentially binds to the appropriately structured tRNA backbone with mature tRNA 5′ and 3′ ends, although it has no preference for intron-containing or intron-less tRNAs [96,100,101,102]. *In vivo* biochemical analyses of the budding yeast Los1 show that both intron-containing pre-tRNAs and spliced tRNAs, regardless of whether they are aminoacylated, assembled into Los1-RanGTP complexes [103]. Consistent with *in vivo* analyses, structural studies show that *S. pombe* Xpo-t interacts with the 3′ end of tRNA but predict that Xpo-t is unlikely to distinguish between charged and uncharged tRNAs [96]. Taken together, all studied exportin-t homologs monitor the common features of tRNA backbone and do not distinguish between intron-containing pre-tRNAs and spliced tRNAs. Interestingly, Lund and Dahlberg show that kinetically end-processing occurs after intron-removal in the nucleus so that exportin-t likely rarely catches intron-containing pre-tRNAs for nuclear export [101]. Despite the fact that pre-tRNA splicing occurs in the nucleus in vertebrates whereas it occurs in the cytoplasm in yeast [104], exportin-t and its homologs are likely to function in both primary nuclear export and re-export of tRNAs.

The vertebrate importin-β family member Exportin-5 (Exp-5) has also been implicated in the nuclear export of tRNA. The genetics data suggested that the yeast Exp-5 homologue, Msn5, is dedicated in the tRNA re-export step for the tRNA encoded by intron-containing genes. *In vivo* biochemical analysis show that Msn5 preferentially assembles with RanGTP and spliced, aminoacylated tRNAs and the specificity of Msn5 for mature tRNAs appears to be due to export complex also assembling with the translation elongation factor, eEF1a. Therefore, Msn5 functions in tRNA nuclear re-export for tRNAs that are encoded by intron-containing genes [103,105].

Although Los1 (exportin-t) and Msn5 (exportin-5) both function in tRNA nuclear export, they cannot be the only nuclear exporters for tRNA as *los1*Δ *msn5*Δ double mutant cells are viable [105,106]. Recent genome-wide studies in yeast report that Crm1 (mammalian exportin-1) may play a role in tRNA nuclear export as cells harboring double mutations of *CRM1* and *LOS1* exhibit synthetic growth defects and mutation of *CRM1* causes altered tRNA nuclear-cytoplasmic distribution [107]. Whether Crm1 directly or indirectly export tRNA to the cytoplasm requires further study.

The tRNA retrograde import process may occur via, at least, two parallel and independent mechanisms. One pathway appears to be mediated by β-importin family member, Mtr10, as cells with deletion of *MTR10* failed to accumulate cytoplasmic tRNAs in the nucleus [105,108]. However, there is no *in vivo* biochemical evidence supporting the RanGTP or RanGDP-dependent interactions of Mtr10 with tRNAs [103]. Mtr10 may function indirectly in tRNA retrograde import. Another pathway appears to be Ran-independent and mediated by the heat shock protein, Ssa2 [109]. Takano *et al.* observed that Ssa2 binds to tRNAs that are poorly folded, suggesting that it has chaperone-like activity for RNA. Cells with deletion of *SSA2* are unable to efficiently import tRNAs into the nucleus upon nutrient starvation, supporting the hypothesis that yeast Ssa2 functions as a carrier for import of cytoplasmic tRNAs into the nucleus [109].

## 8. tRNA Subcellular Dynamics in Response to Nutrient Availability

The tRNA retrograde pathway is responsive to loss of nutrients as cells accumulate tRNA in the nucleus upon amino acids (aa), glucose, or phosphate deprivation [108,109,110,111,112], while cells exhibit an even distribution of tRNA throughout the nucleus and cytoplasm under nutrient replete conditions. The subcellular distribution of tRNAs between the nucleus and the cytoplasm results from the balance among the rates of primary export, nuclear import, and re-export. Retrograde tRNA nuclear import is constitutive [105], thus implicating regulation of nutrient-dependent nuclear accumulation of cytoplasmic tRNAs at the re-export step (Figure 1C). There are multiple possible mechanisms by which tRNA subcellular distribution could be regulated, including nutrient-regulated tethering of tRNAs, as well as regulation of importin-β family members in response to nutrient conditions.

Recent studies have shown that tRNA nuclear re-export is subject to complex regulation as cells utilize distinct mechanisms to respond to aa *vs.* glucose availability [113]. Nuclear-cytoplasmic distributions of Msn5 and Los1 are altered upon glucose deprivation but not aa deprivation. Under fed conditions Los1 and Msn5 are primarily nuclear where they are able to interact with tRNAs and then export them to the cytoplasm. Upon glucose deprivation, Msn5, with kinetics identical to tRNA nuclear accumulation, becomes primarily cytoplasmic and is therefore unable to access nuclear tRNAs to deliver them to the cytoplasm. Redistribution of tRNA exportins upon glucose removal, likely due to collapse of the RanGTP gradient, provides an explanation for nuclear accumulation of imported cytoplasmic tRNAs [113]. Thus, glucose deprivation (but not aa deprivation) causes a rapid (10 min) collapse of the RanGTP gradient, causing tRNAs to accumulate in the nucleus.

Subcellular distributions of the nuclear-cytoplasmic shuttling proteins, Los1 and Msn5, are also affected by carbon source and other stress conditions [114,115,116]. In cells grown with a fermentable sugar as the carbon source, Los1 is located primarily in the nucleus. In contrast, in cells grown in a non-fermentable carbon source, or when the cells are exposed to DNA damaging agents, Los1 is primarily cytoplasmic separated from the tRNAs awaiting nuclear export [114,115,116]. The subcellular distribution of Msn5 is also affected by stress. Msn5 is concentrated in nuclei of unstressed cells, but it is located in the cytoplasm upon exposure to ethanol, heat, starvation, or severe oxidative stress [116,117,118]. Further investigation would be required to understand the crosstalk among tRNA subcellular distribution, the β-importin family, and other stresses.

Upon aa deprivation the subcellular distribution of Los1 and Msn5 resemble fed conditions, thereby implicating other mechanisms independent of the subcellular distribution of Los1 and Msn5 to account for how tRNA re-export responds to aa depletion. As tRNAs can be aminoacylated in the nucleus by the nuclear pool of aminoacyl-tRNA synthetases (aaRS), uncharged tRNA may accumulate in the nucleus when tRNA charging is defective or when cells are deprived for auxotrophic amino acids that they are unable to synthesize [101,105,110,112,119,120,121]. There are two separate studies support the notion that tRNA charging defects inhibit nuclear export of cognate tRNA. First, charging defects in methionyl-tRNA synthetase caused defective nuclear export of cognate tRNA^Met^, however, nuclear export of non-cognate tRNA^Ile^ and tRNA^Tyr^ were not affected [121]. Second, depletion of yeast *THG1*, which is critical for changing of tRNA^His^, results in uncharged tRNA^His^ [122]. *thg1*Δ cells accumulate tRNA^His^ but not tRNA^Tyr^ in the nucleus [122]. Therefore, nuclear aminoacylation status of tRNA affects nuclear export of cognate tRNAs but not the export of non-cognate tRNAs that have been assessed, suggesting that nuclear aminoacylation status of tRNA could provide one regulatory mechanism for cognate tRNA nuclear re-export.

Nuclear accumulation of tRNA upon acute aa removal is rapid and reversible [112]. Upon aa deprivation tRNA^Tyr^, tRNA^Leu^, tRNA^Ile^, tRNA^Met^ and tRNA^His^ accumulate in BY4741 cells [105,108,110,111,112]. Unlike vertebrates, yeasts are able to produce all 20 aa unless they harbor mutations in particular aa biosynthesis pathways. Upon aa deprivation BY4741 cells are able to produce prototrophic amino acids, such as Tyr and Ile, and thus tRNA^Tyr^ and tRNA^Ile^ are charged [112]. However, these charged tRNAs accumulate in the nucleus when cells are depleted of all aa, suggesting that a separate mechanism sensitive to aa availability must play a role in tRNA subcellular dynamics. Ssa2 is a tRNA-binding protein whose deletion compromises nuclear accumulation of tRNAs upon nutrient starvation. Since it has been shown that there is a marginal but reproducible increase in the nuclear pool of Ssa2-FLAG upon aa starvation [109], it is possible that alteration of Ssa2 levels regulate tRNA nuclear import upon aa deprivation. Further investigations are necessary to determine whether other possible mechanisms are involved in regulating tRNA subcellular dynamics, such as aa signaling, alteration of modification status of β-importin or Ssa2, regulation of β-importin or Ssa2 interacting proteins, and tethering of components in the various subcellular compartments.

## 9. tRNA-Derived Fragment as a Regulator in Cellular Process

tRNAs can be cleaved into tRNA-derived ncRNAs in all domains of life and cleavage of tRNAs often occurs during stress conditions [6,123,124,125,126,127,128]. tRNA-derived ncRNAs can be broadly classified into two main groups: tRNA halves and tRNA-derived fragments (tRFs) [123,129]. tRNA halves, which are composed of 30–35 nucleotides derived from either the 5′ or 3′ part of mature tRNAs, are generated under various stress conditions (Figure 1E). When vertebrate tRNAs are exposed to oxidative stress, heat shock, and UV irradiation, angiogenin, a member of the RNase A family, is activated and endonucleolytically cleaves mature tRNAs [130]. Angiogenin may first cleave within the CCA terminus of tRNAs and subsequently cleaves in the anticodon loops of tRNAs, generating 5′ and 3′ tRNA halves [55]. Angiogenin is usually sequestered in the nucleolus or bound to its inhibitor Rnh1 under normal conditions, whereas it is activated and released into the cytoplasm upon exposed to stress. However, the regulation of how angiogenin releases from these cellular compartments is not clear.

In yeast, Rny1, a member of the RNase T2, is responsible for the generation of tRNA halves. Cleavage of yeast tRNA species occurs in response to oxidative stress, methionine starvation, extended growth, and heat, but not in cells undergoing UV stress and glucose starvation [123]. Under normal conditions, yeast Rny1 is localized in the vacuole. Upon exposed to oxidative stress, Rny1 is thought to be released into the cytoplasm, which provides a mechanism by which it could access tRNAs for cleavage [131].

The function of tRNA halves is poorly understood. Certain tRNA halves inhibit translation initiation by displacing the cap-binding complex eIF4F from capped mRNA and inducing the assembly of cytoplasmic stress granules [132,133]. tRNA halves can additionally inhibit stress-induced apoptosis by binding to cytochrome c, therefore protecting cells from apoptosis during osmotic stress [134].

tRNAs can be cleaved at other positions to yield a diverse set of additional tRNA-derived fragments. tRFs are shorter than tRNA halves, ranging from 13 to 32 nt in length (Figure 1F,G). In many organisms, various tRNA-derived ncRNAs are produced from mature tRNAs or their precursor transcripts. Based on their position of origin in pre-tRNAs or mature tRNAs, different types of tRFs are classified as 5′ tRFs, 3′ CCA tRFs, 3′ U tRFs, internal tRFs and 5′ leader-exon tRFs [5,135]. The 5′ tRFs are derived from the 5′ parts of mature tRNAs and are formed by a cleavage in the D loop. 3′ CCA tRFs correspond to 3′ parts of mature tRNAs containing 3′ CCA termini and are formed by cleavage at the T loop. Dicer, angiogenin, and other RNase A family members have been implicated in the generation of the 3′ tRF. The 3′ U tRFs are cleaved by RNaseZ from the 3′ leader sequence of precursor tRNAs (pre-tRNAs) that harbor a stretch of U residues at 3′ end, which is produced upon RNA polymerase III termination. The internal tRFs (itRFs) are derived from a combination of cleavages in the anticodon loop and either D loop or TΨC loop [136]. The 5′ leader-exon tRFs include the 5′ leader sequence of pre-tRNAs and the 5′ part of mature tRNAs [137]. However, the mechanism of formation of 5′ leader-exon tRNAs in not clear.

In higher eukaryotes, some of these tRNA derived tRNA fragments associated with Argonaute proteins and they have emerging roles in gene expression regulation as regulatory RNAs [14,138]. The stress-induced 5′-tRF derived from tRNA^Val^ in the archaeon *Haloferax volcanii* directly binds to small ribosome subunits, and therefore tRF probably fine tunes the rate of protein synthesis to regulate gene expression upon stress conditions [139]. In the pumpkin (*Cucurbita maxima*), tRNA fragments that are found in the phloem sap efficiently inhibit translation in an unspecific manner *in vitro*, indicating that these tRNA fragments may interfere with ribosomal activity [140]. Therefore, accumulating data implicate that fragments as signaling molecules that modulate translation. Furthermore, a tRF derived from the 3′ trailer sequence of human pre-tRNA^Ser^_UGA_ is known to promote cell proliferation [129], suggesting that some tRNA fragments may function beyond translation. Interestingly, recent studies show that paternal diet in mice can alter the levels and/or the modifications of tRNA fragments, mainly derived from 5′ ends of tRNAs, in mature sperm to regulate gene expression of metabolic pathways [141,142].

## 10. Perspectives

tRNA levels are highly regulated and tRNAs are subjected to many post-transcriptional regulatory mechanisms. In budding yeast, 3′ end processing of tRNA is specifically inhibited by certain stress and this stress-responsive 3′ end maturation of tRNA could contribute to fine-tune the levels of functional tRNA in response to cellular conditions. Various sources of stress dynamically shift the population of tRNA modification with unique signature for each type of stress. Discoveries in cell biology have shown that tRNA subcellular traffic is bidirectional between the nucleus and the cytoplasm. Distinct glucose and amino acid dependent mechanisms regulate dynamics of tRNA subcellular distribution upon nutrient deprivation. Production of tRNA fragments serve as intracellular signaling molecules for stress response. Therefore, accumulating data suggest an emerging role of tRNA in stress response and diseases. Although we highlight the possible mechanisms in modulating tRNA pools, the integrated view of how these mechanisms regulate tRNA subcellular dynamics, levels and modification remain unclear. Stress-induced alteration in tRNA modification may affect the function or levels of tRNA in translation or other tRNA-involved biological processes. Whether or how tRNA modification enzymes respond to specific stress to uniquely reprogram tRNA modification levels requires further investigations. Since tRNAs may function as critical regulators in cell proliferation and differentiation, further study of the involvement of tRNA and tRNA fragments in signaling pathways and stress response pathway will lead to new links between tRNA function and other global cellular response systems and will give us insight of how cells respond to the environment.
